# Supplementary material for: Resolving the full spectrum of human genome variation using Linked-Reads
Source: Genome Res. 2019 Apr;29(4):635–45. doi: 10.1101/gr.234443.118 (PMC6442396; doi:10.1101/gr.234443.118)
Supplement: Supplemental Material [file supp_gr.234443.118_Supplemental_longranger-2.2.zip › longranger-2.2.tar.bz2!/lr2.2-public/lib/stan/cmdstan/stan/doc/api/html/gumbel_8hpp_source.html]

Stan: src/stan/prob/distributions/univariate/continuous/gumbel.hpp Source File


|  |  |
| --- | --- |
|  | Stan  2.5.0  probability, sampling & optimization |


- Main Page
- Related Pages
- Namespaces
- Classes
- Files

- File List
- File Members

All Classes Namespaces Files Functions Variables Typedefs Enumerations Enumerator Friends Macros Pages

- src
- stan
- prob
- distributions
- univariate
- continuous

gumbel.hpp

Go to the documentation of this file.

1 #ifndef STAN\_\_PROB\_\_DISTRIBUTIONS\_\_UNIVARIATE\_\_CONTINUOUS\_\_GUMBEL\_HPP

2 #define STAN\_\_PROB\_\_DISTRIBUTIONS\_\_UNIVARIATE\_\_CONTINUOUS\_\_GUMBEL\_HPP

3

4 #include <boost/random/uniform\_01.hpp>

5 #include <boost/random/variate\_generator.hpp>

6

7 #include <stan/agrad/partials\_vari.hpp>

8 #include <stan/math/error\_handling.hpp>

9 #include <stan/meta/traits.hpp>

10 #include <stan/prob/constants.hpp>

11 #include <stan/prob/traits.hpp>

12 #include <stan/prob/internal\_math.hpp>

13 #include <stan/math/functions/value\_of.hpp>

14

15 namespace stan {

16

17  namespace prob {

18

19  template <bool propto, typename T\_y, typename T\_loc, typename T\_scale>

20  typename return\_type<T\_y,T\_loc,T\_scale>::type

21  gumbel\_log(const T\_y& y, const T\_loc& mu, const T\_scale& beta) {

22  static const char\* function = "stan::prob::gumbel\_log(%1%)";

23

24  using std::log;

25  using std::exp;

26  using stan::is\_constant\_struct;

27  using stan::math::check\_positive;

28  using stan::math::check\_finite;

29  using stan::math::check\_not\_nan;

30  using stan::math::check\_consistent\_sizes;

31  using stan::math::value\_of;

32  using stan::prob::include\_summand;

33

34  // check if any vectors are zero length

35  if (!(stan::length(y)

36  && stan::length(mu)

37  && stan::length(beta)))

38  return 0.0;

39

40  // set up return value accumulator

41  double logp(0.0);

42

43  // validate args (here done over var, which should be OK)

44  check\_not\_nan(function, y, "Random variable", &logp);

45  check\_finite(function, mu, "Location parameter", &logp);

46  check\_positive(function, beta, "Scale parameter", &logp);

47  check\_consistent\_sizes(function,

48  y,mu,beta,

49  "Random variable","Location parameter",

50  "Scale parameter",

51  &logp);

52

53  // check if no variables are involved and prop-to

54  if (!include\_summand<propto,T\_y,T\_loc,T\_scale>::value)

55  return 0.0;

56

57  // set up template expressions wrapping scalars into vector views

58  agrad::OperandsAndPartials<T\_y, T\_loc, T\_scale>

59  operands\_and\_partials(y, mu, beta);

60

61  VectorView<const T\_y> y\_vec(y);

62  VectorView<const T\_loc> mu\_vec(mu);

63  VectorView<const T\_scale> beta\_vec(beta);

64  size\_t N = max\_size(y, mu, beta);

65

66  DoubleVectorView<true,is\_vector<T\_scale>::value> inv\_beta(length(beta));

67  DoubleVectorView<include\_summand<propto,T\_scale>::value,

68  is\_vector<T\_scale>::value> log\_beta(length(beta));

69  for (size\_t i = 0; i < length(beta); i++) {

70  inv\_beta[i] = 1.0 / value\_of(beta\_vec[i]);

71  if (include\_summand<propto,T\_scale>::value)

72  log\_beta[i] = log(value\_of(beta\_vec[i]));

73  }

74

75  for (size\_t n = 0; n < N; n++) {

76  // pull out values of arguments

77  const double y\_dbl = value\_of(y\_vec[n]);

78  const double mu\_dbl = value\_of(mu\_vec[n]);

79

80  // reusable subexpression values

81  const double y\_minus\_mu\_over\_beta

82  = (y\_dbl - mu\_dbl) \* inv\_beta[n];

83

84  // log probability

85  if (include\_summand<propto,T\_scale>::value)

86  logp -= log\_beta[n];

87  if (include\_summand<propto,T\_y,T\_loc,T\_scale>::value)

88  logp += -y\_minus\_mu\_over\_beta - exp(-y\_minus\_mu\_over\_beta);

89

90  // gradients

91  double scaled\_diff = inv\_beta[n] \* exp(-y\_minus\_mu\_over\_beta);

92  if (!is\_constant\_struct<T\_y>::value)

93  operands\_and\_partials.d\_x1[n] -= inv\_beta[n] - scaled\_diff;

94  if (!is\_constant\_struct<T\_loc>::value)

95  operands\_and\_partials.d\_x2[n] += inv\_beta[n] - scaled\_diff;

96  if (!is\_constant\_struct<T\_scale>::value)

97  operands\_and\_partials.d\_x3[n]

98  += -inv\_beta[n] + y\_minus\_mu\_over\_beta \* inv\_beta[n]

99  - scaled\_diff \* y\_minus\_mu\_over\_beta;

100  }

101  return operands\_and\_partials.to\_var(logp);

102  }

103

104  template <typename T\_y, typename T\_loc, typename T\_scale>

105  inline

106  typename return\_type<T\_y,T\_loc,T\_scale>::type

107  gumbel\_log(const T\_y& y, const T\_loc& mu, const T\_scale& beta) {

108  return gumbel\_log<false>(y,mu,beta);

109  }

110

111  template <typename T\_y, typename T\_loc, typename T\_scale>

112  typename return\_type<T\_y,T\_loc,T\_scale>::type

113  gumbel\_cdf(const T\_y& y, const T\_loc& mu, const T\_scale& beta) {

114  static const char\* function = "stan::prob::gumbel\_cdf(%1%)";

115

116  using stan::math::check\_positive;

117  using stan::math::check\_finite;

118  using stan::math::check\_not\_nan;

119  using stan::math::check\_consistent\_sizes;

120  using stan::math::value\_of;

121

122  double cdf(1.0);

123  // check if any vectors are zero length

124  if (!(stan::length(y)

125  && stan::length(mu)

126  && stan::length(beta)))

127  return cdf;

128

129  check\_not\_nan(function, y, "Random variable", &cdf);

130  check\_finite(function, mu, "Location parameter", &cdf);

131  check\_not\_nan(function, beta, "Scale parameter", &cdf);

132  check\_positive(function, beta, "Scale parameter", &cdf);

133  check\_consistent\_sizes(function, y,mu,beta,

134  "Random variable","Location parameter",

135  "Scale parameter", &cdf);

136

137  agrad::OperandsAndPartials<T\_y, T\_loc, T\_scale>

138  operands\_and\_partials(y, mu, beta);

139

140  VectorView<const T\_y> y\_vec(y);

141  VectorView<const T\_loc> mu\_vec(mu);

142  VectorView<const T\_scale> beta\_vec(beta);

143  size\_t N = max\_size(y, mu, beta);

144

145  for (size\_t n = 0; n < N; n++) {

146  const double y\_dbl = value\_of(y\_vec[n]);

147  const double mu\_dbl = value\_of(mu\_vec[n]);

148  const double beta\_dbl = value\_of(beta\_vec[n]);

149  const double scaled\_diff = (y\_dbl - mu\_dbl) / beta\_dbl;

150  const double rep\_deriv = exp(-scaled\_diff - exp(-scaled\_diff))

151  / beta\_dbl;

152  const double cdf\_ = exp(-exp(-scaled\_diff));

153  cdf \*= cdf\_;

154

155  if (!is\_constant\_struct<T\_y>::value)

156  operands\_and\_partials.d\_x1[n] += rep\_deriv / cdf\_;

157  if (!is\_constant\_struct<T\_loc>::value)

158  operands\_and\_partials.d\_x2[n] -= rep\_deriv / cdf\_;

159  if (!is\_constant\_struct<T\_scale>::value)

160  operands\_and\_partials.d\_x3[n] -= rep\_deriv \* scaled\_diff / cdf\_;

161  }

162

163  if (!is\_constant\_struct<T\_y>::value) {

164  for(size\_t n = 0; n < stan::length(y); ++n)

165  operands\_and\_partials.d\_x1[n] \*= cdf;

166  }

167  if (!is\_constant\_struct<T\_loc>::value) {

168  for(size\_t n = 0; n < stan::length(mu); ++n)

169  operands\_and\_partials.d\_x2[n] \*= cdf;

170  }

171  if (!is\_constant\_struct<T\_scale>::value) {

172  for(size\_t n = 0; n < stan::length(beta); ++n)

173  operands\_and\_partials.d\_x3[n] \*= cdf;

174  }

175

176  return operands\_and\_partials.to\_var(cdf);

177  }

178

179  template <typename T\_y, typename T\_loc, typename T\_scale>

180  typename return\_type<T\_y,T\_loc,T\_scale>::type

181  gumbel\_cdf\_log(const T\_y& y, const T\_loc& mu, const T\_scale& beta) {

182  static const char\* function = "stan::prob::gumbel\_cdf\_log(%1%)";

183

184  using stan::math::check\_positive;

185  using stan::math::check\_finite;

186  using stan::math::check\_not\_nan;

187  using stan::math::check\_consistent\_sizes;

188  using stan::math::value\_of;

189

190  double cdf\_log(0.0);

191  // check if any vectors are zero length

192  if (!(stan::length(y)

193  && stan::length(mu)

194  && stan::length(beta)))

195  return cdf\_log;

196

197  check\_not\_nan(function, y, "Random variable", &cdf\_log);

198  check\_finite(function, mu, "Location parameter", &cdf\_log);

199  check\_not\_nan(function, beta, "Scale parameter", &cdf\_log);

200  check\_positive(function, beta, "Scale parameter", &cdf\_log);

201  check\_consistent\_sizes(function, y,mu,beta,

202  "Random variable","Location parameter",

203  "Scale parameter", &cdf\_log);

204

205  agrad::OperandsAndPartials<T\_y, T\_loc, T\_scale>

206  operands\_and\_partials(y, mu, beta);

207

208  VectorView<const T\_y> y\_vec(y);

209  VectorView<const T\_loc> mu\_vec(mu);

210  VectorView<const T\_scale> beta\_vec(beta);

211  size\_t N = max\_size(y, mu, beta);

212

213  for (size\_t n = 0; n < N; n++) {

214  const double y\_dbl = value\_of(y\_vec[n]);

215  const double mu\_dbl = value\_of(mu\_vec[n]);

216  const double beta\_dbl = value\_of(beta\_vec[n]);

217  const double scaled\_diff = (y\_dbl - mu\_dbl) / beta\_dbl;

218  const double rep\_deriv = exp(-scaled\_diff) / beta\_dbl;

219  cdf\_log -= exp(-scaled\_diff);

220

221  if (!is\_constant\_struct<T\_y>::value)

222  operands\_and\_partials.d\_x1[n] += rep\_deriv;

223  if (!is\_constant\_struct<T\_loc>::value)

224  operands\_and\_partials.d\_x2[n] -= rep\_deriv;

225  if (!is\_constant\_struct<T\_scale>::value)

226  operands\_and\_partials.d\_x3[n] -= rep\_deriv \* scaled\_diff;

227  }

228

229  return operands\_and\_partials.to\_var(cdf\_log);

230  }

231

232  template <typename T\_y, typename T\_loc, typename T\_scale>

233  typename return\_type<T\_y,T\_loc,T\_scale>::type

234  gumbel\_ccdf\_log(const T\_y& y, const T\_loc& mu, const T\_scale& beta) {

235  static const char\* function = "stan::prob::gumbel\_ccdf\_log(%1%)";

236

237  using stan::math::check\_positive;

238  using stan::math::check\_finite;

239  using stan::math::check\_not\_nan;

240  using stan::math::check\_consistent\_sizes;

241  using stan::math::value\_of;

242

243  double ccdf\_log(0.0);

244  // check if any vectors are zero length

245  if (!(stan::length(y)

246  && stan::length(mu)

247  && stan::length(beta)))

248  return ccdf\_log;

249

250  check\_not\_nan(function, y, "Random variable", &ccdf\_log);

251  check\_finite(function, mu, "Location parameter", &ccdf\_log);

252  check\_not\_nan(function, beta, "Scale parameter", &ccdf\_log);

253  check\_positive(function, beta, "Scale parameter", &ccdf\_log);

254  check\_consistent\_sizes(function, y,mu,beta,

255  "Random variable","Location parameter",

256  "Scale parameter", &ccdf\_log);

257

258  agrad::OperandsAndPartials<T\_y, T\_loc, T\_scale>

259  operands\_and\_partials(y, mu, beta);

260

261  VectorView<const T\_y> y\_vec(y);

262  VectorView<const T\_loc> mu\_vec(mu);

263  VectorView<const T\_scale> beta\_vec(beta);

264  size\_t N = max\_size(y, mu, beta);

265

266  for (size\_t n = 0; n < N; n++) {

267  const double y\_dbl = value\_of(y\_vec[n]);

268  const double mu\_dbl = value\_of(mu\_vec[n]);

269  const double beta\_dbl = value\_of(beta\_vec[n]);

270  const double scaled\_diff = (y\_dbl - mu\_dbl) / beta\_dbl;

271  const double rep\_deriv = exp(-scaled\_diff - exp(-scaled\_diff))

272  / beta\_dbl;

273  const double ccdf\_log\_ = 1.0 - exp(-exp(-scaled\_diff));

274  ccdf\_log += log(ccdf\_log\_);

275

276  if (!is\_constant\_struct<T\_y>::value)

277  operands\_and\_partials.d\_x1[n] -= rep\_deriv / ccdf\_log\_;

278  if (!is\_constant\_struct<T\_loc>::value)

279  operands\_and\_partials.d\_x2[n] += rep\_deriv / ccdf\_log\_;

280  if (!is\_constant\_struct<T\_scale>::value)

281  operands\_and\_partials.d\_x3[n] += rep\_deriv \* scaled\_diff / ccdf\_log\_;

282  }

283

284  return operands\_and\_partials.to\_var(ccdf\_log);

285  }

286

287  template <class RNG>

288  inline double

289  gumbel\_rng(const double mu,

290  const double beta,

291  RNG& rng) {

292  using boost::variate\_generator;

293  using boost::uniform\_01;

294

295  static const char\* function = "stan::prob::gumbel\_rng(%1%)";

296

297  using stan::math::check\_positive;

298  using stan::math::check\_finite;

299

300

301  check\_finite(function, mu, "Location parameter", (double\*)0);

302  check\_positive(function, beta, "Scale parameter", (double\*)0);

303

304  variate\_generator<RNG&, uniform\_01<> >

305  uniform01\_rng(rng, uniform\_01<>());

306  return mu - beta \* std::log(-std::log(uniform01\_rng()));

307  }

308  }

309 }

310 #endif

311

stan::prob::gumbel\_log

return\_type< T\_y, T\_loc, T\_scale >::type gumbel\_log(const T\_y &y, const T\_loc &mu, const T\_scale &beta)

**Definition:** gumbel.hpp:21

traits.hpp

stan::agrad::OperandsAndPartials::to\_var

T\_return\_type to\_var(double logp)

**Definition:** partials\_vari.hpp:138

error\_handling.hpp

stan::length

size\_t length(const T &)

**Definition:** traits.hpp:159

stan::DoubleVectorView

DoubleVectorView allocates double values to be used as intermediate values.

**Definition:** traits.hpp:358

stan::math::check\_finite

bool check\_finite(const char \*function, const T\_y &y, const char \*name, T\_result \*result)

Checks if the variable y is finite.

**Definition:** check\_finite.hpp:52

partials\_vari.hpp

stan::agrad::value\_of

T value\_of(const fvar< T > &v)

Return the value of the specified variable.

**Definition:** value\_of.hpp:16

constants.hpp

stan::prob::gumbel\_ccdf\_log

return\_type< T\_y, T\_loc, T\_scale >::type gumbel\_ccdf\_log(const T\_y &y, const T\_loc &mu, const T\_scale &beta)

**Definition:** gumbel.hpp:234

stan::is\_vector

**Definition:** traits.hpp:83

stan::agrad::OperandsAndPartials

A variable implementation that stores operands and derivatives with respect to the variable...

**Definition:** partials\_vari.hpp:76

traits.hpp

stan::return\_type::type

boost::math::tools::promote\_args< typename scalar\_type< T1 >::type, typename scalar\_type< T2 >::type, typename scalar\_type< T3 >::type, typename scalar\_type< T4 >::type, typename scalar\_type< T5 >::type, typename scalar\_type< T6 >::type >::type type

**Definition:** traits.hpp:406

stan::prob::gumbel\_cdf

return\_type< T\_y, T\_loc, T\_scale >::type gumbel\_cdf(const T\_y &y, const T\_loc &mu, const T\_scale &beta)

**Definition:** gumbel.hpp:113

stan::is\_constant\_struct

Metaprogram to determine if a type has a base scalar type that can be assigned to type double...

**Definition:** traits.hpp:57

stan::math::value\_of

double value\_of(const T x)

Return the value of the specified scalar argument converted to a double value.

**Definition:** value\_of.hpp:24

stan::prob::include\_summand

Template metaprogram to calculate whether a summand needs to be included in a proportional (log) prob...

**Definition:** traits.hpp:35

stan::agrad::OperandsAndPartials::d\_x2

VectorView< double \*, is\_vector< T2 >::value, is\_constant\_struct< T2 >::value > d\_x2

**Definition:** partials\_vari.hpp:83

stan::prob::gumbel\_cdf\_log

return\_type< T\_y, T\_loc, T\_scale >::type gumbel\_cdf\_log(const T\_y &y, const T\_loc &mu, const T\_scale &beta)

**Definition:** gumbel.hpp:181

stan::math::check\_consistent\_sizes

bool check\_consistent\_sizes(const char \*function, const T1 &x1, const T2 &x2, const char \*name1, const char \*name2, T\_result \*result)

**Definition:** check\_consistent\_sizes.hpp:13

stan::max\_size

size\_t max\_size(const T1 &x1, const T2 &x2)

**Definition:** traits.hpp:191

stan::math::check\_positive

bool check\_positive(const char \*function, const T\_y &y, const char \*name, T\_result \*result)

**Definition:** check\_positive.hpp:54

internal\_math.hpp

stan::math::check\_not\_nan

bool check\_not\_nan(const char \*function, const T\_y &y, const char \*name, T\_result \*result)

Checks if the variable y is nan.

**Definition:** check\_not\_nan.hpp:56

value\_of.hpp

stan::agrad::OperandsAndPartials::d\_x1

VectorView< double \*, is\_vector< T1 >::value, is\_constant\_struct< T1 >::value > d\_x1

**Definition:** partials\_vari.hpp:82

stan::agrad::OperandsAndPartials::d\_x3

VectorView< double \*, is\_vector< T3 >::value, is\_constant\_struct< T3 >::value > d\_x3

**Definition:** partials\_vari.hpp:84

stan::agrad::log

fvar< T > log(const fvar< T > &x)

**Definition:** log.hpp:15

stan::VectorView

VectorView is a template metaprogram that takes its argument and allows it to be used like a vector...

**Definition:** traits.hpp:275

stan::agrad::exp

fvar< T > exp(const fvar< T > &x)

**Definition:** exp.hpp:16

stan::prob::gumbel\_rng

double gumbel\_rng(const double mu, const double beta, RNG &rng)

**Definition:** gumbel.hpp:289


---

[ Stan Home Page ]

*© 2011–2014,
Stan Development Team.*
